# High signal intensity on diffusion-weighted magnetic resonance images is a useful finding for detecting early-stage pancreatic cancer

**DOI:** 10.1007/s00261-021-03199-1

**Published:** 2021-07-05

**Authors:** Akira Kurita, Yoshiharu Mori, Yuko Someya, Shigeto Kubo, Shunjiro Azuma, Kosuke Iwano, Satoshi Ikeda, Ryosuke Okumura, Shujiro Yazumi

**Affiliations:** 1 Department of Gastroenterology and Hepatology, Kitano Hospital, Tazuke Kofukai Medical Research Institute, 2-4-20 Ohgimachi, Kita-ku, Osaka, 530-8480 Japan; 2 Department of Diagnostic Imaging, Kitano Hospital, Tazuke Kofukai Medical Research Institute, 2-4-20 Ohgimachi, Kita-ku, Osaka, 530-8480 Japan; 3 Department of Gastroenterology and Hepatology, Rakuwakai Otowa Hospital, 2 Otowachinji-cho, Yamashina-ku, Kyoto, 607-8062 Japan

**Keywords:** Pancreatic cancer, Early diagnosis, Diagnostic modality

## Abstract

**Purpose:**

Early detection of pancreatic ductal adenocarcinoma (PDAC) may improve the prognosis. We evaluated novel imaging findings that may contribute to early detection.

**Methods:**

This single-center, retrospective study enrolled 37 patients with a localized main pancreatic duct (MPD) stricture and no obvious pancreatic mass. All patients underwent endoscopic retrograde cholangiopancreatography and brush sampling with cytology and serial pancreatic juice aspiration cytologic examination via endoscopic naso-pancreatic drainage. Patients with cytology-confirmed malignancy underwent surgical resection. The remaining patients were followed by contrast-enhanced computed tomography (CECT), magnetic resonance imaging (MRI), and endoscopic retrograde cholangiopancreatography.

**Results:**

Twenty patients had confirmed malignancy (cancer group) and 17 did not (non-cancer group). Age, MPD stricture location, and PDAC risk factors were similar, but the sex predominance and symptom rate differed between the two groups. In the cancer group, 17 patients were diagnosed by cytology and three by clinical symptoms. CECT, MRI, and endoscopic ultrasonography (EUS) revealed no solid tumors in either group. CECT revealed no significant differences between groups. Diffusion-weighted MRI revealed significant differences in the signal intensity between groups. EUS detected indistinct and small hypoechoic areas in 70% and 41.2% of patients in the cancer and non-cancer groups, respectively. In the cancer group, 11 were diagnosed with cancer at the first indication, and nine were diagnosed at follow-up; the prognosis did not differ between these two subgroups.ss

**Conclusions:**

High signal intensity in diffusion-weighted MRI may be useful for detecting early-stage PDAC and may be an indication for surgical resection even without pathologic confirmation.

**Clinical trial registration:**

The study was a registered at the University Hospital Medical Information Network (UMIN000039623).

**Graphic abstract:**

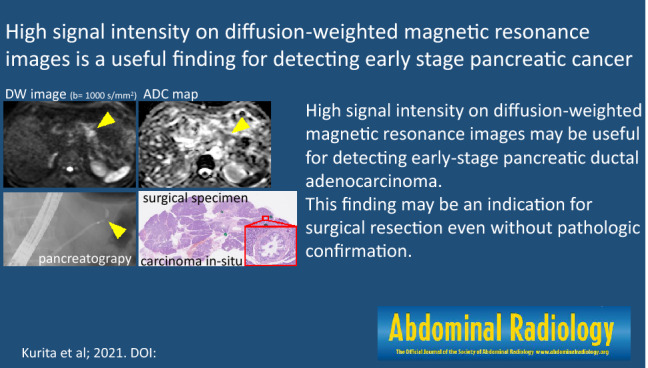

**Supplementary Information:**

The online version contains supplementary material available at 10.1007/s00261-021-03199-1.

## Introduction

Pancreatic ductal adenocarcinoma (PDAC) is a rapidly progressing, highly lethal malignant disease that is difficult to detect in the early stages, due in part to its minimal symptoms early on [1]. PDAC is the fourth leading cause of cancer-related death in the USA and Japan, and the number of deaths is increasing annually worldwide [2]. PDAC is estimated to become the second leading cause of cancer mortality in the USA by 2030 [3]. According to the Japan Pancreatic Cancer Registry in 2012, the 5-year survival rate for patients with stage 0 and stage Ia PDAC in the Union for International Cancer Control (UICC) TNM classification is 85.8% and 68.7%, respectively [4]. The prognosis is highly variable, even between those with stage 0 and Ia. Stage 0 PDAC is noninvasive cancer, namely, carcinoma in situ (CIS). To improve the prognosis of patients with PDAC, early-stage detection is essential. The frequency of stage 0 is approximately 1.7% of all cases and only 25% of these cases are symptomatic [4, 5], which highlights the importance of surveillance for targeted patients during the asymptomatic phase. No effective strategies for detecting early-stage PDAC are established. Recent advances in imaging modalities, including contrast-enhanced computed tomography (CECT), magnetic resonance imaging (MRI), and endoscopic ultrasonography (EUS), allow for the observation of pancreatic CIS [5–12] and the potential detection of indirect imaging findings such as main pancreatic duct (MPD) dilatation, branch duct dilatation, substantial atrophy, and fat substitution of the pancreatic parenchyma for early diagnosis. Screening of patients with risk factors associated with PDAC, such as family history, hereditary pancreatitis, intraductal papillary mucinous neoplasm, diabetes, and smoking [13–19], will also enhance early detection. The aim of the present study was to assess the performance of diffusion-weighted MRI (DWI) for detecting early PDAC in patients with MPD stricture and no obvious pancreatic mass.

## Patients and methods

### Patients

**Fig. 1:**
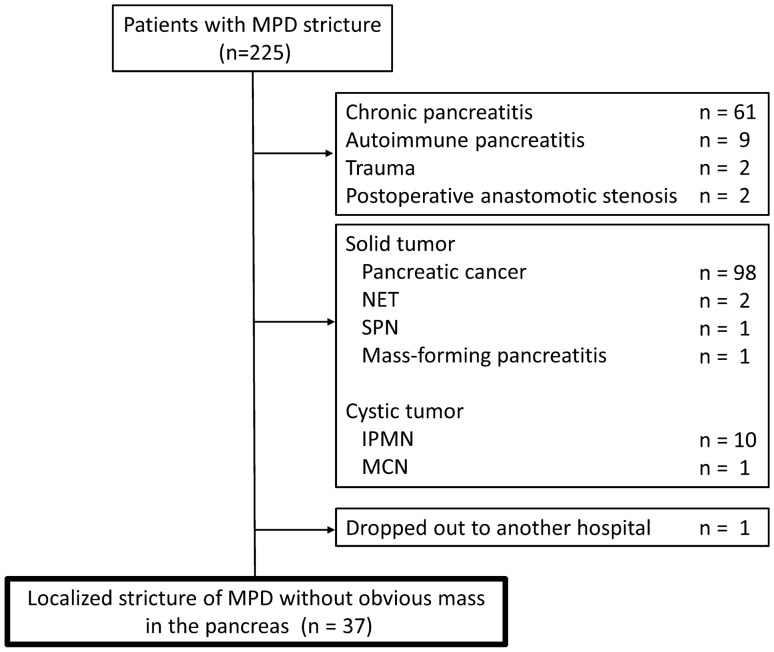
Flowchart of patients throughout the study

Between March 2013 and January 2019, 225 patients were found to have an MPD stricture on images obtained by various modalities, including CECT, MRI, and EUS. Among these 225 patients, those with inflammatory and postoperative diseases, including chronic pancreatitis, autoimmune pancreatitis, trauma, and postoperative anastomotic stricture, were excluded. Those with clear occlusion of the MPD by tumors were also excluded. Finally, 37 patients with a localized MPD stricture and no obvious pancreatic mass were retrospectively enrolled (Fig. 1). All authors had access to the study data and reviewed and approved the final manuscript.

### Study design

This retrospective study was conducted at a single center. The study was approved by the institutional review board and registered at the University Hospital Medical Information Network (UMIN000039623). The patients’ informed consent was waived by the institutional review board.

### Algorithm used in this study

**Fig. 2:**
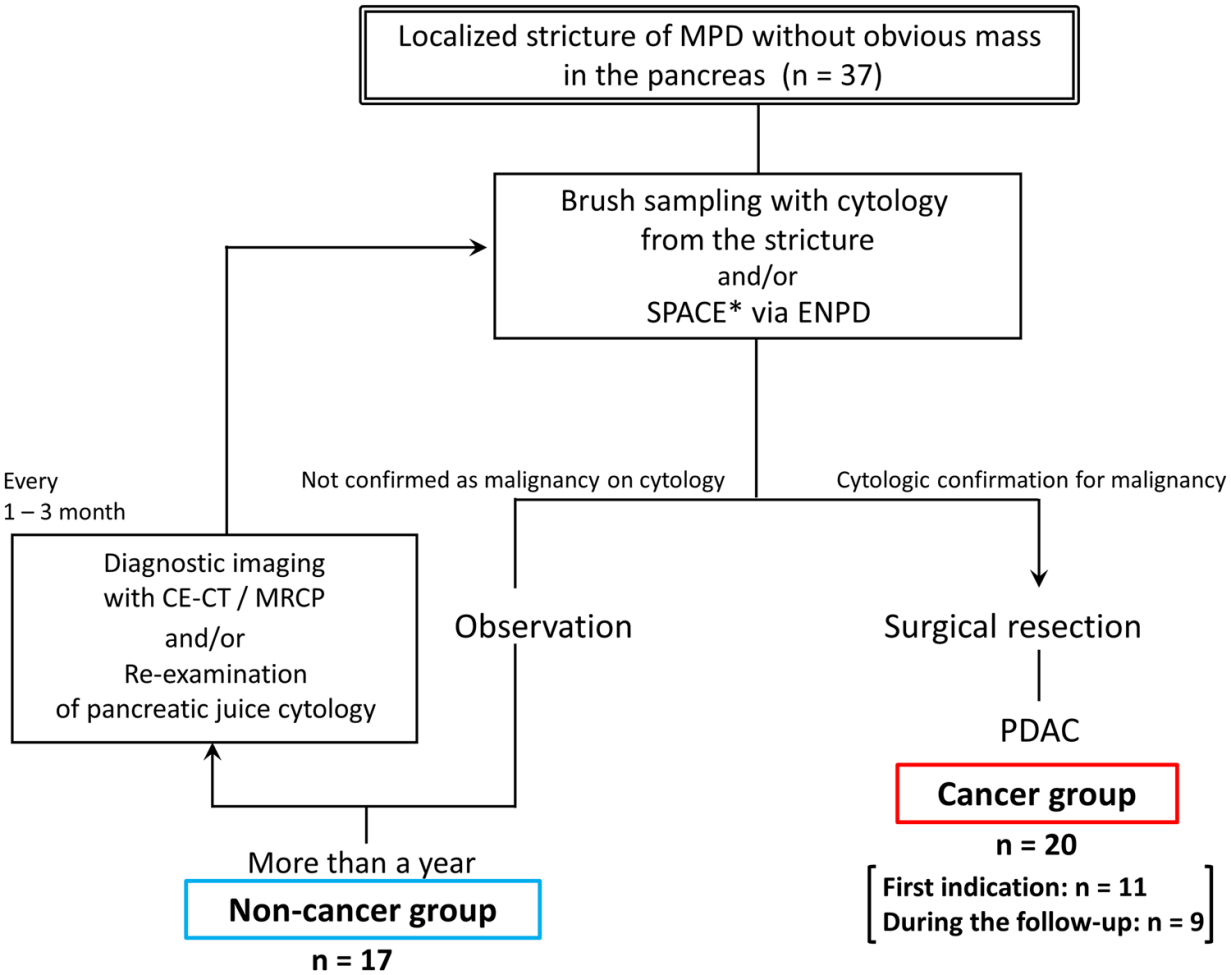
Study algorithm

The study algorithm is shown in Fig. 2. All patients underwent endoscopic retrograde cholangiopancreatography (ERCP), and brush sampling with cytology and serial pancreatic juice aspiration cytologic examination (SPACE) via endoscopic naso-pancreatic drainage (ENPD). Patients diagnosed with malignancy confirmed by cytology underwent surgical resection. The remaining patients were followed by repeating CECT, MRI, and ERCP.

## Techniques and image evaluation

### CT

Multidetector CT (MDCT) examinations were performed using a 64-detector row CT scanner (Aquilion, Toshiba Medical, Japan). The nonionic contrast agent iodixanol (Iopamidol, 300 mg iodine/mL; Fuji Pharma Co., Ltd., Japan) was used at a dose of 1.0–1.9 mL/kg with a 2.2–3.3 mL/s flow rate. The MDCT acquisition parameters were tube voltage, 120 kVp; tube current, auto-adjust; slice thickness, 2.0 mm; reconstruction interval, 0 mm; pitch factor, 0.844. CECT images were obtained at 35, 50, and 180 s after injection of the contrast agent. All CT imaging data were reviewed on a picture archiving and communication system workstation monitor (SYNAPS; Fujifilm Medical Systems, Japan).

### MRI

MRI was performed with an InteraAchieva (Philips Healthcare, Netherlands), which is a 1.5 Tesla whole-body MRI system, and a 16-channel phased array coil as the receiver coil. DWI was performed with respiratory triggering. Apparent diffusion coefficient (ADC) values were calculated by DWI with *b* values of 0 and 1000 s/mm^2^. Frequency-selective fat saturation was used to reduce chemical shift artifacts. Pulse sequence parameters were as follows: repetition time/echo time, 1529/73 ms; field of view, 350 mm; matrix, 96 × 134; number of signals acquired, 6; section thickness, 5 mm; section gap, 0.5 mm; receiver bandwidth, 2301 Hz per pixel; and the acquisition time ranged from 3 to 4 min depending on the patient’s respiratory rhythm. The clinical MRI study also included T1-weighted gradient-echo MRI (183/4.6; flip angle 90°; field of view, 350 mm; matrix, 256 × 206; number of signals acquired, 1; section thickness, 5 mm; section gap, 0.5 mm; acceleration factor, 2), and breath-hold single-shot T2-weighted image (6636/80; flip angle 90°; echo train length, 70; field of view, 350 mm; matrix, 320 × 256; number of signals acquired, 1; section thickness, 5 mm; section gap, 0.5 mm; acceleration factor, 2), and breath-hold 2-dimensional magnetic resonance cholangiopancreatography (10,979/500; flip angle 90°; echo train length, 101; field of view, 280 mm; matrix, 256 × 206; number of signals acquired, 1; section thickness, 5 mm; section gap, − 1.0 mm; acceleration factor, 2). All MRI data were reviewed on the SYNAPS system (Fujifilm Medical Systems).

## EUS procedures

Gastroenterologists well trained in EUS performed the EUS examinations. See the Supplement for details. The pancreas was examined for the presence and size of focal lesions, such as a mass, nodule, small low echoic area, or cyst. Lesions (i.e., nodules or solid masses) were measured in 2 dimensions and characterized by shape, border, echogenicity, heterogeneity, and location. The pancreatic parenchyma and duct were assessed for changes of early chronic pancreatitis according to international consensus statements [20]. The MPD diameter was measured at the proximal and distal sides of the pancreas around the stricture.

### ERCP and cytologic examination procedures

Details of the ERCP procedures are provided in the Supplement. When an MPD stricture was confirmed by pancreatography, brush sampling with cytology was performed and an ENPD tube was placed around the stricture site. SPACE was performed using samples obtained via the ENPD tube at least 6 times in 1 week.

### Image analysis

Two experienced radiologists (K.S. and S.Y.), both with more than 5 years of experience reading abdominal CT and magnetic resonance (MR) images, independently interpreted the CECT and MR images. They were blinded to all clinical information and the final diagnosis. The readers noted the presence or absence of a pancreatic solid mass lesion, MPD stricture with proximal dilation, and pancreatic cyst in all CT and MR images.

The shape of the duct transition (smoothly tapered versus abrupt), maximum diameter of the MPD dilatation, presence of focal fatty changes in the parenchyma, and delayed enhancement were evaluated from CT images according to previous reports [5, 21]. MPD dilatation was considered present when the maximum MPD diameter was ≥ 2.5 mm [22].

The DWI signal intensity was classified on the basis of a qualitative evaluation according to the presence or absence of a hyperintensity lesion compared with the surrounding pancreas. If high signal intensity on diffusion-weighted (DW) images was observed, ADC values were measured for quantitative evaluation. The ADC values were calculated using an operator-defined region-of-interest around the caliber change point. Patients with diffuse high signal intensity on the caudal side due to obstructive pancreatitis (at the distal side of the MPD stricture with a signal intensity difference from the normal-appearing pancreatic tissue) rather than an MPD stricture were not included.

DWI data were reviewed with reference to images from other MRI sequences, such as T1- and T2-weighted images. The presence of obstructive pancreatitis was also evaluated on MR images.

The mean of the values scored by the two radiologists was adopted for quantitative data (i.e., maximum MPD diameter and ADC values). When the CT and MR images were interpreted differently by two evaluators, a final decision was reached by consulting with a third radiologist (O.R.).

EUS images were evaluated by a single gastroenterologist (A.K.) for the presence of a mass, early chronic pancreatitis in the normal parenchyma, and a hypoechoic area around the stricture not recognized as a mass.

### Study definitions

Suspicion of malignancy or malignancy was diagnosed according to the Papanicolaou classification on the basis of cytologic confirmation and patients diagnosed with malignancy underwent surgical resection. The final diagnosis was made following histologic confirmation of PDAC obtained by surgical resection.

The malignant group was defined by histologic confirmation of a surgical specimen. The non-malignant group was defined by the absence of cytologic confirmation of malignancy and followed up for at least 1 year with no remarkable changes observed using either imaging modality. Clinical T factors of pathologic evaluation were determined according to the UICC 8th edition [23].

### Statistical analysis

Categorical parameters were compared using the chi-square test and Fisher exact test, and continuous variables were compared using the Student *t* test and percentages with a 95% confidence interval. Cumulative survival was estimated by Kaplan–Meier analysis, and curves were compared by the log-rank test. All statistical analyses were performed with JMP version 13.0 (SAS Institute, Cary, NC, USA), with *P* < 0.05 considered significant.

## Results

### Patient characteristics

Of the 37 patients enrolled in this study, 20 patients had confirmed malignancy (cancer group) and 17 were classified into the non-cancer group. The demographic data are shown in Table 1. The 2 groups did not differ significantly in age, MPD stricture location, or PDAC risk factors, but the sex predominance and rate of symptoms differed significantly between groups.

**Table 1:** Clinical characteristics of enrolled patients

	Cancer group (*n* = 20)	Non-cancer group (*n* = 17)	*P* value
Sex (male/female)	14/6	5/12	0.02
Median age, year (range)	68.5 (36–86)	70 (49–84)	0.62
Symptoms, *n* (%)	4 (20)	10 (58.8)	0.02
Abdominal pain	4	6	
Back pain	0	2	
Other	0	2	
Serum CEA level, ng/mL, median (range)	2.5 (1.9–4.5)	3.5 (2.0–9.2)	0.84
Serum CA19-9 level, U/mL, median (range)	41.7 (< 2.0–511.2)	7.2 (< 2.0–91.7)	0.07
Follow-up duration, months, median (range)*	9.4 (1.9–43.1)	53.3 (13.8–99.6)	< 0.01
Location of MPD stricture, head/body/tail, *n*	8/4/8	5/4/8	0.80
Risk factors, n (%)**	18 (90)	11 (64.7)	0.11
DM	3	1	0.61
Smoking	13	2	< 0.01
IPMN	5	7	0.48
Chronic pancreatitis	0	0	–
Heavy alcohol consumption	8	3	0.17
Obesity	7	3	0.29
Family history of PDAC	2	1	1.00

*MPD* main pancreatic duct, *DM* diabetes mellitus, *IPMN* intraductal papillary mucinous neoplasm, *PDAC* pancreatic ductal adenocarcinoma

^*^Observation period of cancer group was the duration between the initial diagnosis and the time of cancer diagnosis of followed 9 patients

^**^Some cases had multiple risk factors

### Findings of each diagnostic imaging modality

Table 2 shows the CECT, MRI, and EUS findings. CECT revealed no obvious solid mass in either group. The proximal dilated MPD diameter and number of focal fatty changes of the pancreatic parenchyma, delayed enhancement, existence of a pancreatic cyst, and the MPD stricture type were not significantly different between the two groups.

**Table 2:** Imaging modalities and findings

Modalities and findings	Cancer group (*n* = 20)	Non-cancer group (*n* = 17)	*P* value
CECT			
Obvious solid mass	0	0	–
MPD stricture with proximal dilation	20 (100)	17 (100)	1.00
MPD stricture shape, abrupt/smooth	13/7	11/6	1.00
Proximal MPD diameter, mm, median (range)	5.0 (2.5–8.5)	4.3 (2.5–10.0)	0.79
Focal fatty changes of parenchyma	11 (55)	8 (47.1)	0.75
Delayed enhancement	9 (45)	8 (47.1)	1.00
Pancreatic cyst	10 (50)	10 (58.8)	0.74
MR			
Obvious solid mass	0	0	–
MPD stricture with proximal dilation	20 (100)	17 (100)	1.00
Pancreatic cyst	10 (50)	10 (58.8)	0.74
Obstructive pancreatitis	7 (35)	3 (17.6)	0.29
High intensity on DW	13 (65)	1 (5.9)	< 0.01
EUS			
Obvious solid mass	0	0	–
Small hypoechoic area	14 (70)	7 (41.2)	0.10
Early chronic pancreatitis	10 (50)	4 (23.5)	0.17

Values are *n* (%) unless otherwise defined

*CECT* contrast-enhanced computed tomography, *MPD* main pancreatic duct, *MR* magnetic resonance, *DW* diffusion weighted, *EUS* endoscopic ultrasonography

MRI revealed no obvious solid mass in either group. Areas of high signal intensity in DWI differed significantly between the two groups (*P* < 0.01). One false positive case in the non-cancer group on the basis of the DW image had a mean ADC value of 1.27, but close follow-up was selected because no malignancy was confirmed on cytologic examination and no changes were observed over 51.6 months.

For quantitative analysis, the ADC was measured in 13 patients in the cancer group and 1 patient in the non-cancer group (Table 3). ADC values did not differ significantly between groups (*P* = 0.95).

**Table 3:** Quantitative analysis of diffusion-weighted image

Case No	Pathologic T stage	Reader 1	Reader 2
1	Tis	1.65	1.62
2	Tis	1.78	1.43
3	Tis	1.39	1.05
4	Tis	1.67	1.94
5	Tis	1.02	1.02
6	T1b	1.21	1.18
7	T1b	1.22	1.03
8	T1c	1.37	1.28
9	T1c	2.02	1.68
10	T1c	1.29	1.34
11	T1c	1.40	1.48
12	T2	1.50	1.28
13	T3	1.24	1.22
14	Non-cancer group	1.26	1.27

ADC values are in units of × 10^−3^mm^2^/s

EUS images showed no obvious solid mass in either group. An indistinct and small hypoechoic area, however, was detected in 70% of patients in the cancer group and 41.2% in the non-cancer group.

**Fig. 3:**
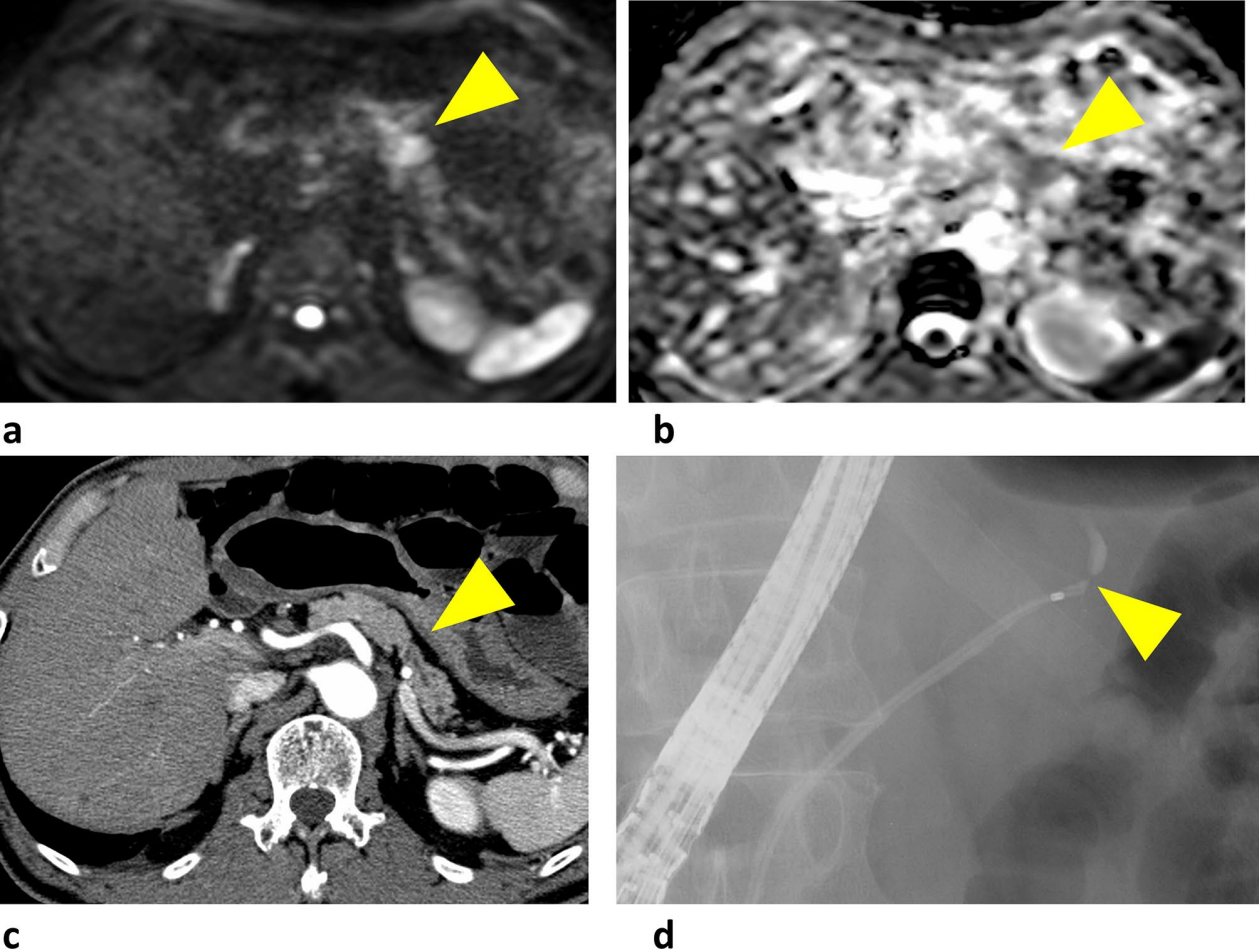
**a** High signal intensity around the stricture in the diffusion-weighted image (arrowhead). **b** apparent diffusion coefficient values by 2 readers were 1.65 and 1.62 (× 10^−3^mm^2^/s), respectively. **c** Focal pancreatic parenchyma atrophy and main pancreatic duct stricture without a visible mass on computed tomography (arrowhead). **d** pancreatography shows a high-grade pancreatic duct stricture in the pancreatic tail

**Fig. 4:**
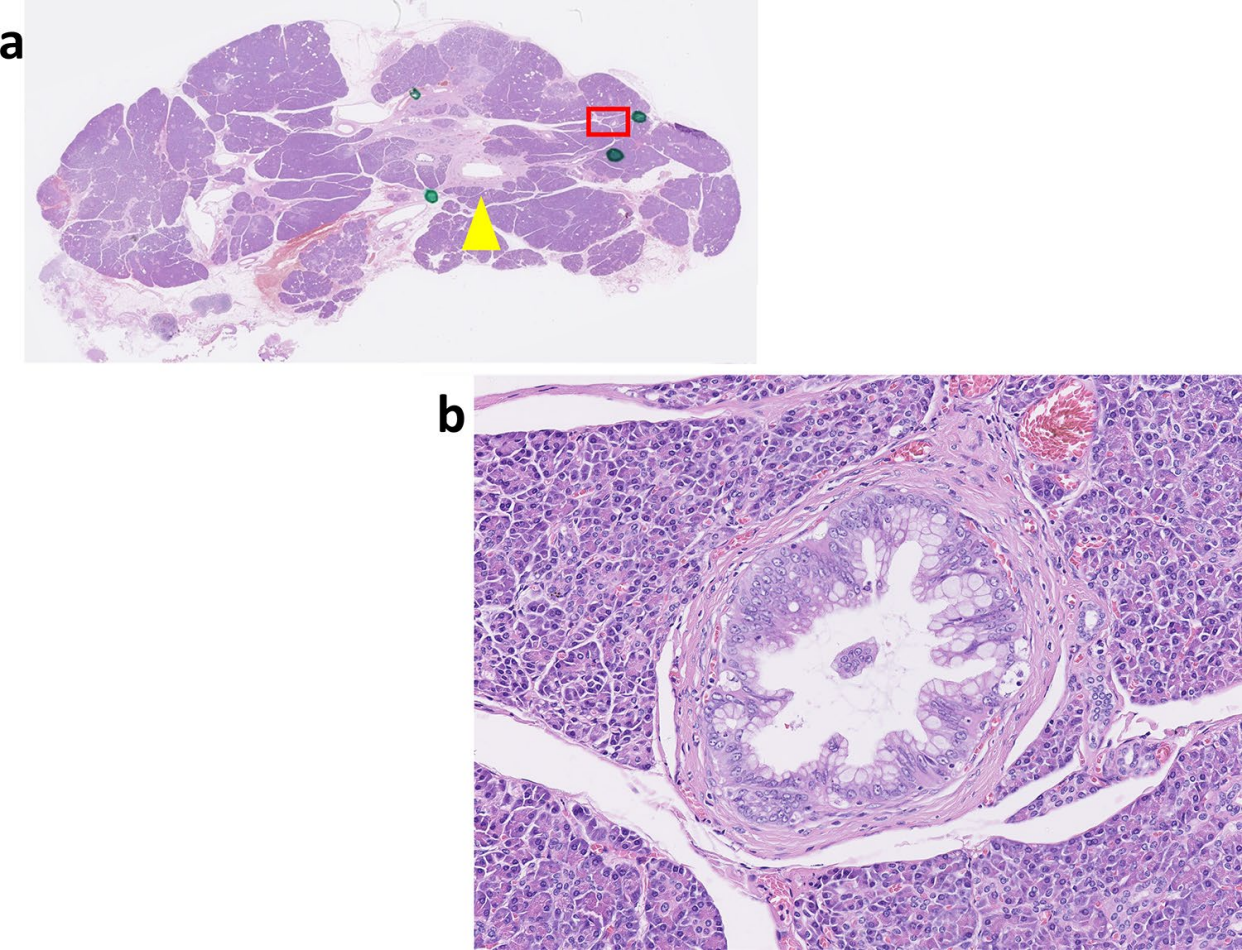
Resected pancreas with carcinoma in situ. High-grade pancreatic intraepithelial neoplasia in the periphery of the pancreas (hematoxylin & eosin, orig. mag. 40X). **a** Main pancreatic duct is indicated by the arrowhead. **b** Area in the red box

A typical case is shown in Fig. 3. Focal pancreatic parenchymal atrophy and an MPD stricture without an obvious solid mass were visible on CT. DWI high signal intensity and an elevated ADC value were observed around the stricture. ERCP showed an MPD stricture in the tail of the pancreas, and the results of brush sampling with cytology were suspicious for malignancy. Distal pancreatectomy was performed and pathologic examination of the resected specimen indicated CIS (Fig. 4).

### Detailed characteristics of the cancer group

**Fig. 5:**
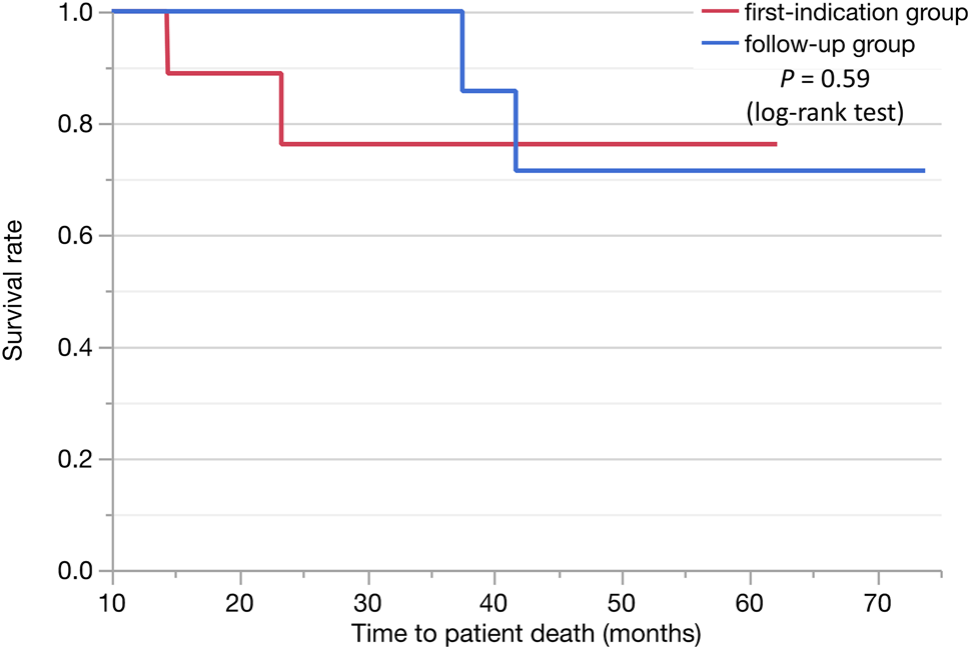
Comparison of the prognosis for patients diagnosed at the first indication and those diagnosed during follow-up

Surgical resection was performed in all patients in the cancer group. The T factors of pathologic evaluation are shown in Table 4. The timing of the surgical resection varied, even for those with CIS. Of the 20 patients in the cancer group, 11 were diagnosed with PDAC at the first indication and 9 were diagnosed during the follow-up. The median follow-up period for the diagnosis of PDAC was 9.4 (range: 1.9–43.1) months in the follow-up group (Table 1). The prognosis did not differ between patients diagnosed at the first indication and those diagnosed during the follow-up (*P* = 0.59; Fig. 5).

**Table 4:** Detailed staging factors of cancer group

Pathologic T stage	*N* (%)	Timing of PDAC diagnosis, initial/follow-up	High intensity on DW, *n* (%)
Tis	7 (35)	3/4	5 (71.4)
T1a (≤ 5 mm)	2 (10)	2/0	0
T1b (5 mm < tumor < 10 mm)	2 (10)	1/1	2 (100)
T1c (1–2 cm)	5 (25)	2/3	4 (80)
T2 (2 cm < and ≤ 4 cm)	3 (15)	2/1	1 (33.3)
T3 (4 cm <)	1 (5)	1/0	1 (100)

Among the 20 patients in the cancer group, 17 were diagnosed with malignancy by cytologic analysis. Of these 17 cases, 6 were diagnosed by both brush cytology and SPACE, 5 were diagnosed by brush cytology alone, and 6 were diagnosed by SPACE alone. As to the other 3 patients of the 20, the result of repeated cytologic analysis suggested malignancy, and although the results were inconclusive, the patients with clinically diagnosed with malignancy based on their cytology result and the shape of the pancreatic duct stricture, and underwent surgery during the follow-up period.

## Discussion

This study is the first to describe DWI high signal intensity as a useful finding for detecting early-stage PDAC. Importantly, areas of high signal intensity in DW images were even observed in 71.4% of patients with CIS.

Multiple centers worldwide have developed pancreatic screening programs to detect early-stage PDAC in select populations of high-risk individuals (HRIs) [17–19, 24–29]. According to the International Cancer of the Pancreas Screening Consortium, HRIs are defined as those with a defined genetic syndrome and those without a diagnosed syndrome but with familial pancreatic cancer [18, 19, 25]. The incidence of PDAC is also increasing in the general population and most cases of PDAC are not found in HRIs, emphasizing the need for nonselective screening. Based on current evidence targeting HRIs, MRI and EUS should be first-line tests for pancreatic surveillance. EUS and MRI, however, are not adequate for detecting small PDACs, including CIS, with a diameter less than 1 cm, and there are few reports of early-stage PDAC detection due to the lack of effective imaging modalities and biomarkers for small PDAC and CIS [24, 30–32].

Some researchers focusing on small PDACs report that the most abnormal findings obtained using various diagnostic imaging modalities to detect early-stage PDAC are indirect findings, such as MPD dilatation or retention cysts, with no direct findings of PDAC [5, 33, 34]. These findings suggest that attention should be focused on specific populations at increased risk for PDAC and the detection of indirect findings on diagnostic images in asymptomatic cases, and that further examination in such cases is important for early diagnosis of PDAC.

Several reports suggest that high signal intensity on DW images compared with surrounding tissue or benign lesions indicates the presence of PDAC [35–43]. Some tumors of a certain size show hyperintensity on DW images, whereas small tumors, especially those < 1 cm, do not. Some researchers report that localized fatty changes of the pancreatic parenchyma observed in CT images may be a feature of CIS [5, 34]. Our findings, however, demonstrated that localized fatty changes were not a specific feature of CIS because 47.1% of patients in the non-cancer group showed this change, and no significant difference was detected between the cancer and non-cancer groups. Localized pancreatitis with infiltration of inflammatory cells, fibrosis, and fatty infiltration is frequently observed in the parenchyma around CIS and atypical epithelium [34]. Thus, our finding that even CIS appeared as an area of hyperintensity indicates that hyperintensity does not reflect the tumor itself, but rather the surrounding inflammation and fibrosis [44, 45]. Supplementary Figure shows a case of CIS. High signal intensity on DW images indicated inflammation and fibrosis around the CIS lesion by pathologic analysis.

The most important factor in diagnosing early-stage PDAC is the confirmation of malignancy by cytologic examination. Previous reports indicate that cytologic examination of the pancreatic juice and brush sampling has a sensitivity of 33%–80% [46–51]. False-negative cases, however, remain a problem. In our study, cytologic diagnosis was positive in 11 of 20 patients (55%) in the cancer group at the initial examination, and 3 cases in the follow-up group underwent surgical resection without cytologic confirmation of malignancy. These 3 patients were clinically diagnosed with malignancy and surgery was performed after follow-up periods of 7.9, 30.2, and 43.1 months. All 3 cases were in the pT1 stage. Five patients in the follow-up group, other than four cases with CIS, might have been diagnosed at an earlier stage if the initial cytology-based diagnosis had been accurate. To increase the sensitivity of cytologic examination, some studies have analyzed the usefulness of repeated samplings evaluated by SPACE and secretin injection, but the sensitivity is at most 80% [51]. Therefore, high signal intensity on DW images, as reported here, is a highly useful finding for diagnosing early-stage PDAC, leading to a recommendation for surgical resection without cytologic confirmation.

The present study has several limitations. First, the sample size was relatively small and retrospective studies are more prone to bias than prospective studies. A prospective study with a larger number of patients should be performed to confirm our findings. Second, PDAC may also arise later in the non-cancer group, although no PDAC was detected in those patients during a median follow-up duration of 53 months. A longer follow-up period is needed to rule out this possibility. Third, areas of inflammation, such as obstructive pancreatitis, may also appear as high signal intensity on DW images. Patients with diffuse high signal intensity on the caudal side rather than an MPD stricture were excluded, but it is difficult to exclude them completely because pancreatitis sometimes appears mottled and heterogeneous. Because the images were interpreted by two independent radiologists, however, the possibility of obstructive pancreatitis was minimized. Fourth, some patients in the cancer group were found to be in an advanced stage, which may be due to the rapid progression of PDAC. To address this possibility, the waiting time until surgery should be reduced, and ideally a test should be developed that can identify tumors at the cellular level.

In conclusion, patients with an MPD stricture should undergo MR imaging and evaluation of areas with high signal intensity on DW images for detection of early-stage PDAC. Surgical resection should be considered for some patients showing high signal intensity areas on DW images, even without prior pathologic confirmation of PDAC.

## Supplementary Information

Below is the link to the electronic supplementary material.

Supplementary file1 (TIF 6265 kb)

Supplementary file2 (DOCX 19 kb)

## Abbreviations

ADC: Apparent diffusion coefficient
CECT: Contrast-enhanced computed tomography
CIS: Carcinoma in situ
CT: Computed tomography
DW: Diffusion weighted
DWI: Diffusion-weighted MRI
ENPD: Endoscopic naso-pancreatic drainage
ERC: Endoscopic retrograde cholangiopancreatography
EUS: Endoscopic ultrasonography
HRI: High-risk individual
IRB: Institutional review board
MDCT: Multidetector computed tomography
MPD: Main pancreatic duct
MR: Magnetic resonance
MRI: Magnetic resonance imaging
PDAC: Pancreatic ductal adenocarcinoma
SPACE: Serial pancreatic juice aspiration cytologic examination
UICC: Union for International Cancer Control
