# Supplementary figures and images for: High signal intensity on diffusion-weighted magnetic resonance images is a useful finding for detecting early-stage pancreatic cancer
Source: Abdom Radiol (NY). 2021 Jul 5;46(10):4817–27. doi: 10.1007/s00261-021-03199-1 (PMC8435518; doi:10.1007/s00261-021-03199-1)

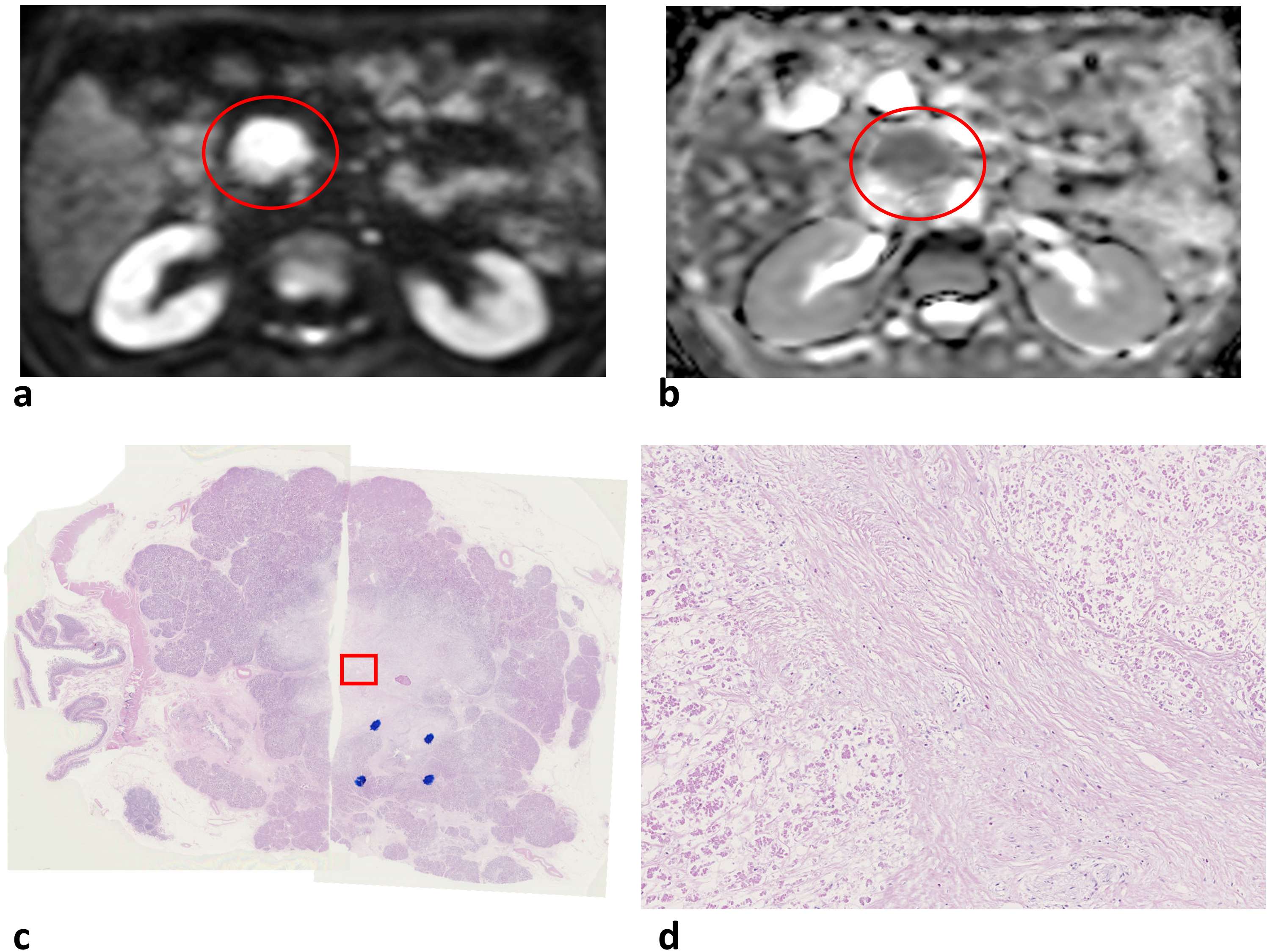

Supplement: Supplementary file 1 — Supplementary file1 (TIF 6265 kb) [file 261_2021_3199_MOESM1_ESM.tif]
