# Supplementary material for: High signal intensity on diffusion-weighted magnetic resonance images is a useful finding for detecting early-stage pancreatic cancer
Source: Abdom Radiol (NY). 2021 Jul 5;46(10):4817–27. doi: 10.1007/s00261-021-03199-1 (PMC8435518; doi:10.1007/s00261-021-03199-1)
Supplement: Supplementary file 2 — Supplementary file2 (DOCX 19 kb) [file 261_2021_3199_MOESM2_ESM.docx]

**Supplemental Methods**

**EUS procedures**

Gastroenterologists well trained in EUS performed the EUS examinations using a radial echoendoscope (GF-UE260-AL5, Olympus Optical Corp Ltd, Japan) or linear array echoendoscope (GF-UCT 260, Olympus Optical Corp Ltd, Japan). All examinations were performed in patients under conscious sedation with midazolam.

**ERCP and cytologic examination procedures**

Gastroenterologists well trained in ERCP performed the ERCP using a side-viewing duodenoscope (TJF-260V or TJF-Q290V, Olympus Medical Systems Co. Ltd, Japan) in patients under conscious sedation with diazepam and pethidine hydrochloride, and continuous pulse oximetry.

**Supplemental Figure Legends**

Supplementary Figure. MR imaging and resected pancreas with CIS. A, high signal intensity around the pancreatic head in the DW image. B, the ADC images showed hypointensity. C and D, inflammation and fibrosis cells occupied around the CIS lesion.

**Supplemental Table**

Supplementary Table. Magnetic resonance (MR) parameters

| Sequence | TR/TE (ms) | Flip angle (°) | Section thickness (mm) | Matrix size | Field of view (mm) | Acquisition time (s) | No. of excitations |
| --- | --- | --- | --- | --- | --- | --- | --- |
| T1W-2D GRE | 183/4.6 | 90 | 5 | 256x206 | 350 | 15 | 1 |
| BH-SS-T2WI | 6636/80 | 90 | 5 | 320x256 | 350 | 13 | 1 |
| DW imaging | 1529/73 | 90 | 5 | 80x112 | 350 | 150 | 6 |
| BH-2D-MRCP | 10979/500 | 90 | 5 | 256x206 | 280 | 22 | 1 |

T1W-2D, T1-weighted two dimensional; GRE, gradient echo; BH-SS-T2WI, breath-hold single-shot T2-weighted image; DW, diffusion-weighted; MRCP, MR cholangiopancreatography; TR, repetition time; TE, echo time
